# Individual differences in migratory behavior shape population genetic structure and microhabitat choice in sympatric blackcaps (*Sylvia atricapilla*)

**DOI:** 10.1002/ece3.825

**Published:** 2013-10-01

**Authors:** Gregor Rolshausen, Gernot Segelbacher, Claudia Hermes, Keith A Hobson, H Martin Schaefer

**Affiliations:** 1 Redpath Museum and Department of Biology, McGill University859 Sherbrooke St. W., Montreal, Quebec, H3A 0C4, Canada; 2 Faculty of Biology, Department of Evolutionary Biology and Animal Ecology, University of FreiburgHauptstrasse 1a, 79104, Freiburg, Germany; 3 Department of Wildlife Ecology and Management, University FreiburgTennenbacher Str. 4, D-79106, Freiburg, Germany; 4 Environment Canada11 Innovation Blvd., Saskatoon, Saskatchewan, S7N 3H5, Canada

**Keywords:** Blackcap, bottleneck, micro-evolution, microhabitat choice, migratory connectivity, migratory divide, spatial isolation, stable isotopes

## Abstract

In migratory birds, traits such as orientation and distance are known to have a strong genetic background, and they often exhibit considerable within-population variation. How this variation relates to evolutionary responses to ongoing selection is unknown because the underlying mechanisms that translate environmental changes into population genetic changes are unclear. We show that within-population genetic structure in southern German blackcaps (*Sylvia atricapilla*) is related to individual differences in migratory behavior. Our 3-year study revealed a positive correlation between individual migratory origins, denoted via isotope (*δ*^2^H) values, and genetic distances. Genetic diversity and admixture differed not only across a recently established migratory polymorphism with NW- and SW-migrating birds but also across *δ*^2^H clusters within the same migratory route. Our results suggest assortment based on individual migratory origins which would facilitate evolutionary responses. We scrutinized arrival times and microhabitat choice as potential mechanisms mediating between individual variation in migratory behavior and assortment. We found significant support that microhabitat choice, rather than timing of arrival, is associated with individual variation in migratory origins. Moreover, examining genetic diversity across the migratory divide, we found migrants following the NW route to be genetically more distinct from each other compared with migrants following the traditional SW route. Our study suggests that migratory behavior shapes population genetic structure in blackcaps not only across the migratory divide but also on an individual level independent of the divide. Thus, within-population variation in migratory behavior might play an important role in translating environmental change into genetic change.

## Introduction

In several migratory species, hybrid zones between recently diverged taxa are located on migratory divides, which are areas where populations with different migratory behavior meet (Bensch et al. 2009; Brelsford and Irwin 2009; Ruegg et al. 2012). Thus, migratory behavior has been considered an important factor influencing assortative mating and, consequently, population divergence and even speciation (Price 2007; Liedvogel et al. 2011). However, mechanisms how migratory behavior can lead to divergence are not well understood, mainly because the genetic structure of migratory populations is typically unknown. It is thus important to link within-population variation in migratory traits with population genetic structure.

In particular, it has been hypothesized that migratory connectivity (i.e., the linkage between breeding, stopover, and wintering areas) can affect the genetic structure of populations and the ability of migratory species to evolve in response to changing selective pressures (Webster et al. 2002). For example, if individuals from a breeding population overwinter in different regions, there will be substantial genetic variation for migratory traits (such as distance and orientation) in that population. Nonrandom aggregation on the breeding grounds based on similarities in individual wintering origins would then facilitate ongoing population divergence, whereas random structuring would prevent divergence (Helbig 1996; Pulido et al. 1996). While previous studies analyzed whether migratory behavior contributed to population divergence (Bensch et al. 2009; Rolshausen et al. 2009), to our knowledge, no study has attempted to ask how individual migratory routes, and distances contribute to within-population structuring.

In this study, we examine whether individual variation in wintering origins predicts population genetic structure of sympatric blackcaps (*Sylvia atricapilla*) in southern Germany. Contingent upon our findings, we then analyse whether the observed genetic structure of the population is explicable by temporal and spatial isolation in arrival times and microhabitat choice of territorial blackcaps. Earlier work showed that migratory orientation and distance in blackcaps, a species that migrates alone, are not learned but have a strong genetic component (Berthold 2000). Furthermore, rapid micro-evolutionary changes of its migratory phenology have been observed (Berthold et al. 1992; Pulido and Berthold 2010). In particular, the recently established migratory polymorphism in southern German blackcaps (Berthold et al. 1992) is known to facilitate reproductive isolation and drive population divergence in sympatry (Bearhop et al. 2005; Rolshausen et al. 2009). Thus, because it is known that individual differences in migratory traits influence mating behavior (Bearhop et al. 2005), we do not focus on this topic but rather specifically ask how within-population variation in individual claw tip stable isotope (*δ*^2^H) values, a proxy for the birds' wintering area (Bearhop et al. 2005), relates to within-population genetic structuring, arrival times, and microhabitat choice. We further scrutinize within-population variation in *δ*^2^H and microsatellites to ask how the migratory polymorphism in our study population affects genetic variability on either side of the migratory divide. Uncovering this link between individual migratory behavior and population genetic structure will help the evaluation of the potential for micro-evolutionary responses to contemporary changes in selective regimes, for instance advancing spring phenology due to climate change.

Previous studies used stable-hydrogen isotope values (*δ*^2^H) to directionally assign blackcaps to either one of the two main wintering origins of southern German blackcaps. Those origins were northwestern (NW) migrants wintering in Great Britain or south-western (SW) migrants wintering in the Mediterranean (Bearhop et al. 2005; Rolshausen et al. 2009). Assignments were based on the fact that *δ*^2^H values of foodwebs determining blackcap claw isotope values were driven primarily by latitudinal gradients in amount-weighted long-term patterns of average *δ*^2^H in precipitation for Europe (Bowen et al. 2005). On average, more southern wintering populations were expected to arrive on German breeding grounds with higher *δ*^2^H values than more northern populations such as those wintering in the U.K. (Bearhop et al. 2005). As these probability assignments were a priori referenced with tissue *δ*^2^H distributions from prespecified wintering grounds, they necessarily resulted in a dichotomous “either-or” outcome and therefore discounted individual variation in wintering origins within each of the regions. However, the wintering range of blackcaps is large and ringing recoveries reveal that it is continuous rather than dichotomous covering substantial areas throughout Western Europe, including Belgium, northern Germany, France, southern and northern Spain (Snow and Perrins 1997; Mokwa 2009). Furthermore, contemporary shifts in wintering ranges and a reduction in migratory activity in response to changing climate are well documented for central European migrants, including the blackcap (Fiedler 2003). In a recent study, Pulido and Berthold (2010) showed that migratory activity within a south-west migrating population of southern German blackcaps decreased significantly over 14 years arguing that these changes presumably involve a micro-evolutionary response to climate-induced directional selection for shorter migration routes favoring earlier arrival and earlier breeding.

We suggest that such particularly rapid genetic adjustment would be facilitated by a link between individual wintering origin and genetic structuring within breeding populations mediated by nonrandom pairing according to individual migratory strategies. This hypothesis has not yet been investigated.

To examine the hypothesis that the population genetic structure of southern German blackcaps is affected by differences in individual wintering origins within a population, we used *δ*^2^H values in the birds' claw tips as a continuous proxy for wintering area. While the pattern of *δ*^2^H values in keratin of songbirds in Europe is expected to primarily reflect a northeast–southwest axis for origins of individuals (Bowen et al. 2005; Hobson 2011), populations differing in *δ*^2^H values can be assumed to derive from different, albeit unknown, wintering origins (Hobson 2005; Studds et al. 2012). Also, within-population variance in *δ*^2^H values among songbirds from the same location is of the order of 9–12‰ (Hobson et al. 2012). With these caveats in mind, we used blackcap tissue *δ*^2^H values as a conservative means of exploring linkages between genetic structure and potentially different wintering origins. We considered detectable trends in any relationship between blackcap tissue *δ*^2^H values and population genetic structure as strong evidence linking isolation on wintering grounds to nonrandom mating on sympatric breeding grounds.

Unlike previous studies (Bearhop et al. 2005; Rolshausen et al. 2009, 2010), we do not use a priori information to assign individuals to prespecified wintering origins but calculate pairwise similarities in *δ*^2^H values across the whole population (to account for a continuous wintering range) and perform an uninformed cluster analysis to portion individuals based on *δ*^2^H similarities (*δ*^2^H clusters). We then partitioned the overall genetic diversity in our dataset on the basis of variation in blackcap tissue *δ*^2^H values. Assuming that individual differences in migratory behavior contribute to population genetic structuring, we predicted (1) pairwise genetic distances to increase with pairwise geographical distances between wintering areas and (2) pairwise genetic distances within *δ*^2^H clusters to be lower than the mean pairwise genetic distance across the whole study population. We (3) examined whether spatio-temporal isolation of blackcaps contributed to the observed genetic structure of sympatric blackcaps. To our knowledge, this is the first study that investigates population genetic structure in relation to migratory behavior on an individual level.

## Methods

### Field procedures and sampling

We caught blackcaps with mist nets upon their spring arrival on southern German breeding grounds in Radolfzell (47°45′N 08°59′E) in 2006 and in Freiburg (48°00′N 07°51′E) in 2007 and 2008. The Freiburg site was chosen because of logistical reasons and was also surveyed for habitat analyses in 2010 and 2011 (see below). In each year, we captured birds from mid-March on, when the first migrants arrived from their wintering quarters to mate on their breeding grounds, and caught birds every day from early morning to noon until the end of April. Blackcaps were caught within an area of 50 ha in a deciduous forest with mist nets using tape recordings of their song as a decoy. Each morning we intensively patrolled for singing activity and captured all newly singing males as well as nonsinging individuals within this area. Because of this procedure, we considered the first day a bird was caught as a proxy for arrival date and we calculated a dayscore as the difference in arrival relative to the start of the field season for statistical analysis (days from 15th March). Our proxy for arrival date reflects accurately arrival in the study area. However, we cannot exclude that an individual had already arrived a few days before in an area outside the study area. All individuals were marked with a standard aluminum ring (additional color coded rings in case of habitat examination, see below), sexed, aged (Shirihai et al. 2001), and weighed (digital balance ±0.1 g precision) before obtaining blood samples (50–75 μL) and claw tip samples. Claw tips were then analyzed for their individual stable isotope signature (^2^H/^1^H ratio, denoted as *δ*^2^H) using the comparative equilibration method described in detail by Wassenaar and Hobson (2003) and through the use of calibrated keratin isotope reference materials (see: Rolshausen et al. 2010 for methodological details).

Signals based on tissue *δ*^2^H measurements have been used successfully to infer wintering origins of migratory passerines, and blackcaps in particular, based on a large-scale latitudinal stable isotope gradient across central Europe (Bearhop et al. 2005; Bowen et al. 2005; Rolshausen et al. 2010). Along this gradient *δ*^2^H values are higher in the north-northwest and lower in the south (Bowen et al. 2005, see also: http://www.waterisotopes.org). Individual *δ*^2^H values entered our analyses as a continuous variable and were used as a proxy for (north–south) geographical distance between wintering origins (Webster et al. 2002; Hobson 2005). As our dataset potentially includes both locally breeding migrants and migrants caught “en route,” we used wing morphology to assign individuals as either local southern German breeding birds or long-distance migrants on their migration to more northerly breeding grounds. The assignment was based on probability density functions (see Rolshausen et al. 2010 for details) using Fiedler (2005) as well as our own measurements as reference data. From the 195 individuals that were analyzed in this study, 142 were considered as locally breeding middle-distance migrants based on their wing morphology. In the result section, we provide test statistics for this specific subset along with our main results for the whole dataset.

Nuclear DNA was extracted from blood samples using the DNeasy Blood and Tissue Kit (Qiagen, Hilden, Germany). We used the following microsatellites for the genetic analysis: Syl1, Syl2, Syl3, Syl4, Syl5, Syl6, Syl7, Syl8, Syl9, Syl10 (for details on markers and PCR conditions see: Segelbacher et al. 2008; Rolshausen et al. 2009). In total, we analyzed isotope signatures and genomic data for 195 birds from the three consecutive study years.

### Habitat analyses

To investigate whether microhabitat choice of blackcaps on the breeding grounds is related to individual variation in wintering origins, we analyzed the habitat structure for 36 breeding territories observed in 2010 and 2011 at our field sites in Freiburg. At the beginning of the respective breeding season, a male was considered territorial for a specific area if he was repeatedly spotted in that area for a minimum of 10 consecutive days, displayed territorial behavior (i.e., calls and singing), and/or was accompanied by a female. The margins of a territory were either defined as a 20 m radius from the center where the male was spotted most of the time or they were inferred from male's behavioral patterns (i.e., antagonistic interactions with other males). The territory size estimated by both measures did not differ ( C. Hermes, G. Segelbacher and H. Martin Schaefer, unpubl. data).

In mid June, each territory was surveyed for its vegetation structure by recording the variables described in Table 1; some of them were previously identified as being important for the establishment of territories in blackcaps (Hoi-Leitner et al. 1995). The data were then analyzed with a principal component analysis (PCA) to examine the distribution of individuals in an ordination diagram (i.e., habitat space). To examine whether microhabitat choice is associated with wintering origins, we (1) use the individual scores on the first two habitat principal components (PC1 and PC2) to test for a linear relationship with the respective individual *δ*^2^H values and (2) use a generalized additive model (GAM) to fit a smooth response surface of *δ*^2^H values over the limits of the ordination biplot. We include the latter procedure to additionally test for a combined nonlinear association between the habitat ordination axes (“habitat space”) and individual *δ*^2^H values.

**Table 1:** List of the variables recorded for the microhabitat analysis along with a brief description of how they were recorded. Loadings correspond to correlation coefficients between principal components and variables, respectively (importance cutoff limit: 0.2). Numbers 1–9 correspond to the arrows 1–9 in Fig. 5

		Loadings		
		
Habitat variable	Description	PC1	PC2	PC3
1. Lower shrubbery layer (1.5–3 m)	% of vegetation in this layer	**0.559**	−0.038	**0.272**
2. Upper shrubbery layer (3–5 m)	% of vegetation in this layer	**0.516**	**0.277**	**0.253**
3. Crown layer (above 5 m)	% of vegetation in this layer	0.035	**0.593**	−**0.341**
4. Nettle (Urtica dioica) covering	% of vegetation covered by nettles	−0.054	−**0.324**	−**0.339**
5. Bramble (Rubus sp.) covering	% of vegetation covered by brambles	−0.059	−0.010	0.116
6. Number of ivy (Hedera helix) trees	Number of trees covered in ivy	0.189	**0.528**	−**0.318**
7. Blooming shrubs	% of blooming individuals in shrubbery layer	**0.390**	−**0.209**	0.121
8. Blooming herbs	% of blooming individuals in herbaceous layer	**0.424**	−**0.323**	−**0.315**
9. Disturbance	% of the territory that is disturbed (e.g., proximity to highways, noise, walking paths)	**0.214**	−0.197	−**0.634**

### Individual genetic distance, diversity, and admixture

Individual genetic distance matrices based on the 10 microsatellites were calculated using the software package microsat (http://hpgl.stanford.edu/projects/microsat). The following genetic distance measures were calculated in our study: Reynolds *Theta* (Reynolds et al. 1983), Slatkins *Rst* (Slatkin 1995), and Goldsteins *deltaMu* ([δμ]^2^, Goldstein et al. 1995). Furthermore, we calculated a pairwise distance matrix based on individual *δ*^2^H values to infer individual differences in wintering origin within the studied population. Correlations between pairwise geographical distances and pairwise genetic distances were then analyzed with Mantel's nonparametric test (Mantel 1967). In addition, to correct for pseudo-replication due to pairwise comparisons, we analyzed the relation between genetic distance and geographical distances using a linear mixed model with geographical distance as fixed effect and individuals as a random structure. Significance for the fixed effect was based on (1) the posterior distribution of the model parameter obtained via MCMC simulations (*n* = 10,000) and (2) loglikelihood comparisons between the full model and the reduced model (excluding *δ*^2^H as a fixed effect).

To examine the relation between individual wintering origins and population genetic structuring, we applied a nonhierarchical cluster analysis to our *δ*^2^H variable that separates migrants based primarily on the north–south geographical distance between individual wintering origins. The appropriate number of “*δ*^2^H clusters” was objectively evaluated on the basis of the cumulative explained variance among clusters. This method recommends choosing that number of clusters so that further splitting does not provide more relevant information (i.e., “elbow criterion”). Respective clusters were then analyzed for deviations of their mean within-cluster genetic distance from the overall mean genetic distance to ask whether the cluster-intern genetic structure was more homogenous than that of the overall population structure of the dataset (i.e., indicating assortative pairing). This was tested by permutating the composition of respective clusters, while keeping cluster sizes constant. We analyzed between-cluster differences in individual genetic diversity using two different measures: mean individual heterozygosity and mean *d*² (Coulson et al. 1998). Inbreeding coefficients (*F*_IS_) between *δ*^2^H clusters were calculated with *F*_STAT_ (Goudet 1995).

The software BOTTLENECK (Piry et al. 1999) was used to scrutinize *δ*^2^H clusters for deviations from expected heterozygote excess relative to allelic diversity across all microsatellite loci. These tests within the *δ*^2^H clusters were performed on the basis of the proposed mutation models for microsatellites: the infinite alleles model (IAM), the stepwise mutation model (SMM), and the two-phase model (TPM: 70% SMM, 30% IAM). Studies on avian microsatellite evolution suggest that the SMM/TPM models are most appropriate (Primmer and Ellegren 1998; Beck et al. 2003). However, a proportion of our markers are compound markers with imperfect sequence motifs (Segelbacher et al. 2008), more likely to evolve under the IAM (Estoup et al. 1995). We therefore included the SMM, TPM, and IAM in our analysis. Note that the heterozygosity excess compares observed and expected heterozygosity and should not be confused with an excess of heterozygotes (Cornuet and Luikart 1996).

A Bayesian admixture model analysis was conducted on population structure using the software STRUCTURE 2.3 (Pritchard et al. 2000) and the therein implemented model of informative priors (LOCPRIOR, Hubisz et al. 2009). The LOCPRIOR model included the *δ*^2^H clustering and was run for *k* = 2 and *k* = 3 population clusters. These *k*-values were chosen according to (1) an evaluation of the most likely *k* for our dataset (without a priori information) using STRUCTURE and the Δk criterion (Evanno et al. 2005; yielded *k* = 2, for details see: Rolshausen et al. 2009) and (2) the most informative clustering of the *δ*^2^H variable (yielded *k* = 3, Fig. 2). STRUCTURE was run for 10 separate MCMC simulations over 50,000 burn-ins with 100,000 repeats for each *k,* and the different runs were then merged using CLUMPP (Jakobsson and Rosenberg 2007). Based on the likelihood assignment of the admixture model, we calculated (1) individual confidence coefficients (Δ*p*_r_) as the residuals of the highest assignment score to the random assignment value (*p*_r,2clusters_ = ½ and *p*_r,3clusters_ = ⅓) and (2) the overall percentage of each simulated genetic cluster within the respective *δ*^2^H clusters. These two simple measures allow a proportionate comparison of the genetic clusters within the *δ*^2^H clusters.

All results reported for the genetic distance are based on Reynolds *Theta* distance but do not change qualitatively if Slatkins *Rst* (Slatkin 1995) or Goldsteins *deltaMu* (Goldstein et al. 1995) are employed. We provide the alternative test statistics along with our results. All basic statistical procedures were performed using R (R Development Core Team 2010).

## Results

**Figure 1:**
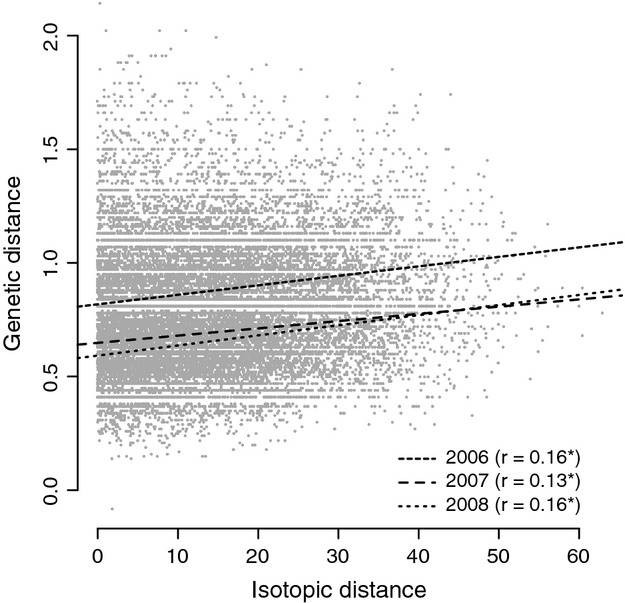
Correlation between individual genetic distance and delta distance (based on *δ*^2^H values). The scatterplot shows individual pairwise distances from all three study years (2006–2008, note that 2006 birds and 2007/2008 birds were not caught at the same site), and the lines denote the positive correlations for each year separately. Numbers in parentheses depict the respective Pearson correlation coefficients (*: all *P*≪0.01).

We found a significant positive correlation between the individual pairwise genetic distance (*Theta*) and the individual pairwise *δ*^2^H distances in claw stable isotope values (*r* = 0.13, *P* = 0.001, *n* = 195). The mixed model incorporating individuals as a random structure also revealed a significant effect of *δ*^2^H distance on genetic distance (Model estimates: Intercept = 0.760, *δ*^2^H = 0.005, MCMC simulation: *P*-value_*δ*2H_ = 0.001; reduced vs. full model: ΔAIC = 510.29, *P*-value<0.0001). The relatively low correlation coefficient is typical for individual pairwise comparisons illustrating substantial genetic and geographical variation in overwintering locations in our blackcap population. Similar results were found when we (1) based the test on alternative genetic distance measures (Slatkins Rst: *r* = 0.10, *P* = 0.003; Goldsteins [δμ]^2^: *r* = 0.10, *P* = 0.001, Mantel's test with 1000 permutations, *n* = 195), (2) excluded potential long-distance migrants from our dataset (*r* = 0.15, *P* = 0.002, *n* = 142), and (3) partitioned our dataset according to study year and/or site (Fig. 1). To account for a possible bias from an underlying population structure caused by the NW–SW migratory divide in our data (Rolshausen et al. 2009), we additionally ran Mantel's test on the SW migrants only. This test yielded comparable population structuring within SW migrants (*r* = 0.10, *P* = 0.033). We (2) repeated the test on 500 randomly chosen subsets comprising 100 individuals from our dataset, respectively. Here, we found that 80% of all 500 Mantel tests were significant (*P* < 0.05) and 93% were marginally significant (*P* < 0.1) with a mean test statistic comparable to our original results (*r* ± SE=0.13 ± 0.002). Isotope distances were not related to individual distances in arrival times of birds on their breeding grounds (*r* = 0.03, *P* > 0.7, Mantel's test with 1000 permutations) suggesting that individual birds from distinct wintering regions did not differ strongly in arrival times.

**Figure 2:**
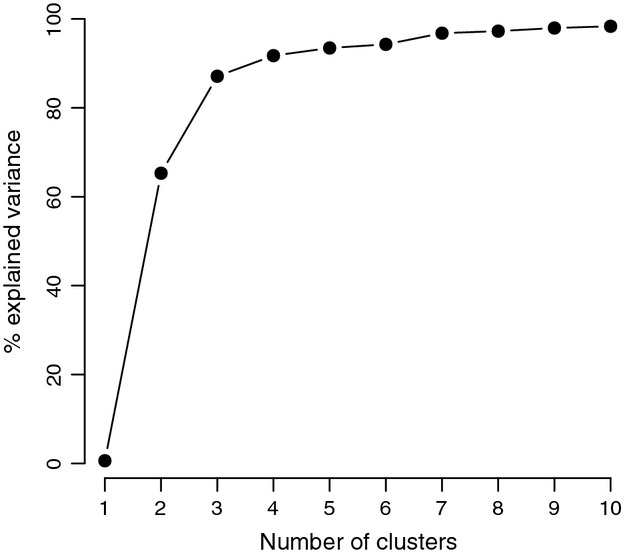
Evaluation of best clustering based on partitioning around medoids (pam) applied to the *δ*^2^H variable. The graph depicts the course of the ratio of within-group variances by the total variance along an increasing number of clusters. We chose three clusters to be the optimal representation of the data with ∼90% of the total variance explained.

To analyse the relation of migratory origin and the underlying population structure, we partitioned the *δ*^2^H variable into separate clusters. The evaluation of the most informative partition according to the cumulative explained variance yielded three wintering origins illustrated as *k* = 3 *δ*^2^H clusters as the fitting selection (Fig. 2): one *δ*^2^H cluster containing birds presumably from more northerly wintering areas (N migrants, *δ*^2^H cluster 3: *n* = 55, mean *δ*^2^H = −91.2‰) and two *δ*^2^H clusters containing birds presumably from more southerly wintering quarters (S-migrants), separated according to their approximate distance to the breeding grounds (farther: *δ*^2^H cluster 1: *n* = 52, mean *δ*^2^H = −59.6‰; nearer: *δ*^2^H cluster 2: *n* = 88, mean *δ*^2^H = −74.4‰).

**Figure 3:**
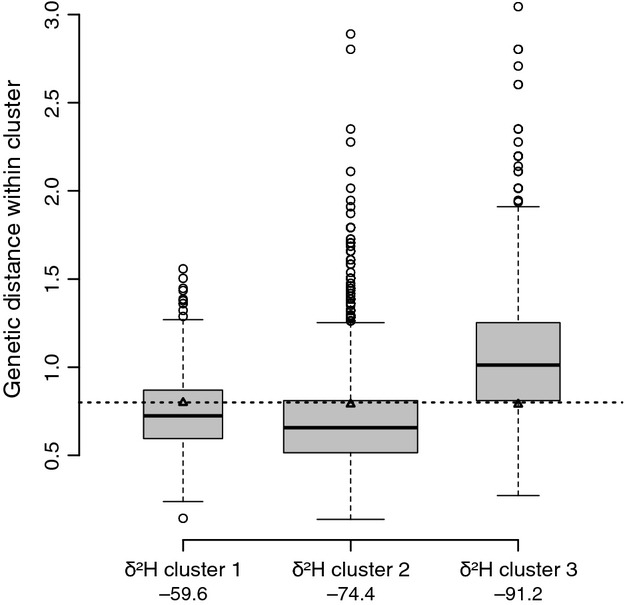
Individual pairwise genetic distances within *δ*^2^H clusters obtained from partitioning the *δ*^2^H variable. The *x*-axis denotes the respective clusters with their mean *δ*^2^H values (‰) and the *y*-axis denotes the genetic distance as Reynolds *Theta*. The dotted horizontal line marks the overall mean genetic distance for the dataset, and triangles denote the respective mean distances within clusters after randomized cluster assignment (1000 permutations). Mean genetic distances within clusters were significantly different from either the permutation means or the overall mean (cluster 1 [*n* = 52]: *P* = 0.015, cluster 2 [*n* = 88]: *P* = 0.004, cluster 3 [*n* = 55]: *P* = 0.000, overall mean: 0.785, *P*-values obtained over 1000 permutations).

A particularly interesting result was that the mean individual genetic distances among birds overwintering at northern latitudes were higher compared with birds that overwintered south of the breeding grounds (*δ*^2^H cluster 3 vs. *δ*^2^H cluster 1 and 2, Fig. 3). Further, *δ*^2^H clusters tended to differ in their mean within-genetic distance from the mean within-genetic distance obtained by permutation of the entire population. The northerly migrants were significantly more distant from each other than the southerly wintering migrants which were less distant from each other compared with the permutation mean (*δ*^2^H cluster 1: *P* = 0.015, *δ*^2^H cluster 2: *P* = 0.004, *δ*^2^H cluster 3: *P* = 0.000, permutation test, Fig. 3). Both measures of genetic diversity, mean heterozygosity, and mean d² were significantly lower in N migrants (*δ*^2^H cluster 3) compared with the other *δ*^2^H clusters (*δ*^2^H cluster 3 vs. *δ*^2^H cluster 1 and 2: all *P* < 0.01, comparisons between *δ*^2^H clusters 1 and 2: *P* > 0.1). The inbreeding coefficient (*F*_IS_) was higher for the N migrants and not different among the two S-migrating clusters (*F*_IS *δ*_^2^_H cluster 3_: 0.45; *F*_IS *δ*_^2^_H cluster 2_: 0.27; *F*_IS *δ*_^2^_H cluster 1_: 0.22; *P* ≤ 0.10, pairwise Wilcoxon tests with adjusted *P*-values). No heterozygote excess in either *δ*^2^H cluster was found when testing under the assumptions of the SMM (*P* > 0.80), whereas there was a significant heterozygote excess found under TPM and IAM assumptions (all *P* ≤ 0.05, Wilcoxon signed rank test), indicating no clear evidence for a recent bottleneck in any cluster.

**Figure 4:**
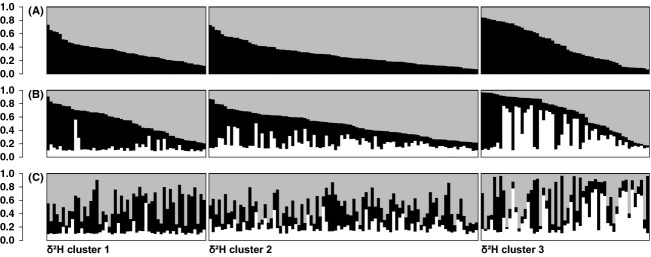
Coefficients of admixture for each individual estimated using STRUCTURE. Individuals in their respective *δ*^2^H cluster are represented by columns, and individual admixture coefficients are shown as proportions of different colors in each column. A: admixture model assuming two underlying populations; B and C: admixture model assuming three underlying populations. Barplots A and B align individuals within *δ*^2^H clusters according to their admixture coefficients, barplot C aligns individuals in ascending order of their *δ*^2^H values.

STRUCTURE simulations assuming *k* = 2 genetic clusters yielded no significant differences in admixture among the three *δ*^2^H clusters (Fig. 4A). Individual confidence coefficients (Δp_r_) did not differ between the *δ*^2^H clusters (all *P* ≥ 0.10), nor did the overall proportions of each genetic cluster in the *δ*^2^H clusters (genetic cluster 1: 66% in *δ*^2^H cluster 1; 68% in *δ*^2^H cluster 2; 56% in *δ*^2^H cluster 3). However, when simulating *k* = 3, we found significant differences in Δp_r_ and overall proportions of genetic clusters between *δ*^2^H clusters (Fig. 4B). Blackcaps wintering in more northern areas, represented by *δ*^2^H cluster 3, had significantly higher Δp_r_ scores compared with *δ*^2^H clusters 1 and 2 (all *P* < 0.010), indicating a more confident assignment of individuals from that *δ*^2^H cluster to either of the three simulated populations. We also found marginal differences in Δp_r_ scores between *δ*^2^H clusters 1 and 2 (*P* = 0.100, pairwise Wilcoxon test with adjusted *P*-values). The percentage represented by the third genetic cluster within each of the *δ*^2^H clusters increased from 14% in cluster 1 to 21% in cluster 2, to 36% in cluster 3 (Fig. 4B) and assignment scores to the third genetic cluster correlated significantly with individual *δ*^2^H values (*r* = 0.48, *P* < 0.001, *n* = 195, Fig 4C). Given the unbiased approach of the LOCPRIOR admixture model (Hubisz et al. 2009), the reported differences between *δ*^2^H clusters suggest asymmetric admixture in relation to overwintering origins.

**Figure 5:**
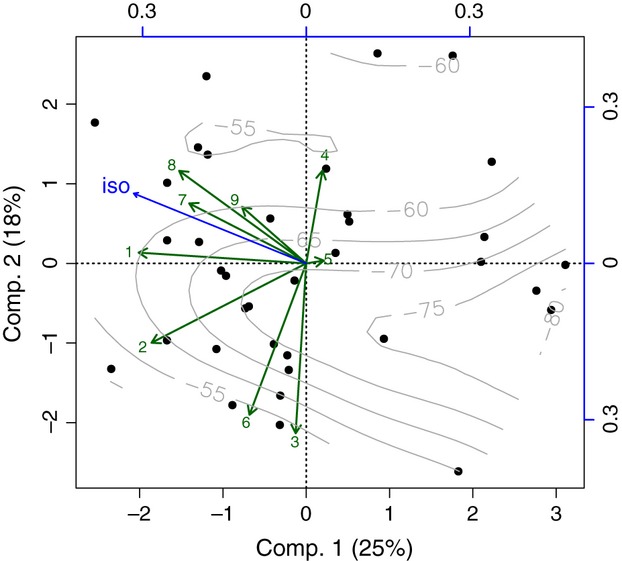
Habitat choice ordination biplot with *δ*^2^H variable response surface overlain in gray and the surface was fitted using a generalized additive model approach. The arrows 1–9 depict the respective habitat variables (see Table 1). The iso-arrow depicts the direct biplot projection for the *δ*^2^H variable onto the ordination and indicates the significant association with Comp. 1 but not with Comp. 2.

The analysis of the microhabitat choice of blackcaps resulted in three main principal components accounting for a total of 60% of variation (PC1:25%, PC2:18%, PC3:17%) in the birds' distribution (Fig. 5). Five of nine habitat variables were considered important loadings (correlation coefficient >0.2, Table 1) for PC1 basically describing vegetation density (in lower and upper shrubbery layers) and the amount of blooming shrubs and herbs. PC2 was mainly affected by vegetation density in the crown layer, the density of ivy (*Hedera helix*), and presence of nettles (*Urtica dioica*), and PC3 was mainly affected by disturbance and density in the crown layer and presence of nettles (Table 1).

Using linear models, we found a significant correlation of individual PC1 scores with *δ*^2^H values (cor.*r* = −0.323, *P* = 0.050, Fig. 5) but no relation between *δ*^2^H values and PC2 (cor.r = 0.120, *P* = 0.467) and a marginal relation with PC3 (cor.r = −0.268, *P* = 0.102). However, assuming a nonlinear relation, the generalized additive model fitted onto the first two PCs indicated a significant association between individual *δ*^2^H values and the main individual variation in occupancy of the habitat space (GAM fit: F = 2.24, *P* = 0.047, Fig. 5). Taken together, both results suggest that – independent of the underlying assumption – blackcaps establish territories in different habitats depending on their individual wintering origins.

## Discussion

### Migratory connectivity and genetic structuring

Here, we show that individual migratory behavior within a population contributes to the genetic structure of sympatric southern German blackcaps. In particular, birds with more similar *δ*^2^H values were genetically more closely related to each other (pairwise differences at neutral loci) than to birds wintering at more distant locations. Initially, it seems that the overall correlation (*r* = 0.13) in the pairwise comparisons among 195 individuals was weak. However, this only shows that there is substantial genetic variation within blackcap populations which is consistent with other studies on migratory birds (Bensch et al. 2009; Prochazka et al. 2011). A comparable coefficient was recently reported for a population of Darwin's finches where individual beak morphology correlated with genetic distance at *r* = 0.13 (De León et al. 2012). Indeed, the pattern we report is likely to be biologically relevant because it was consistently found in three consecutive years, at two different study sites (Fig. 1), and also occurred for >80% of 500 randomly drawn subsets of our dataset and for the subset excluding potential long-distance migrants. Taken together, these results indicate that similarity in wintering origin influences genetic distances even on shared breeding grounds. This conclusion mirrors that from Bearhop et al. (2005). However, our results indicate that the influence of wintering origin on mating decisions not only occurs dichotomously along the migratory divide (Bearhop et al. 2005; Rolshausen et al. 2009) but also to a similar extent within the same migration route (SW route) on an individual level throughout the population. Hence, an important novel insight from our study is that the influence of wintering origin on population structuring is not solely attributable to the two migratory directions in Southern German blackcaps (NW and SW), but may be more prevalent in migratory birds in general. Thus, rapid translations of environmental change into micro-evolutionary changes, such as the genetic reduction in migratory activity in blackcaps due to climate-driven selection (Pulido and Berthold 2010), could be facilitated by genetic structuring within populations according to individual migratory routes and distances.

The proximate mechanisms mediating the effect of migratory connectivity on the genetic structure of sympatric populations are currently not well understood. Our habitat analyses reveal a significant relation between the characteristics of individual territories and the tissue *δ*^2^H values of males occupying these territories (Fig. 5). This link between breeding habitat choice and wintering origin suggests that where a bird overwinters may have profound influence on which habitat it chooses for breeding and that this in turn may affect reproductive output (see also Norris and Taylor 2006; Norris and Marra 2007). For example, blackcaps might colonize breeding territories that are more similar to their wintering quarters. While this conjecture has not yet been investigated, carry-over effects are more generally important at the individual level and can translate variation in migratory behavior into assortative mating on shared breeding grounds (Reudink et al. 2009). Our study showed no effect of temporal isolation because differences in arrival times were not significantly related to the respective wintering origins of individual blackcaps inferred from tissue *δ*^2^H values. This result supports previous analyses from our population (Rolshausen et al. 2010) and contrasts those of more eastern blackcap populations where assortative mating according to migratory route seems to be more prevalent (Bearhop et al. 2005). Spatial rather than temporal separation might therefore contribute to explain the effect of migratory origins on the genetic structure of the sympatric populations in south-western Germany. Furthermore, it is currently unknown whether postzygotic barriers additionally contribute to the observed genetic structure of blackcaps. In general, postzygotic barriers are deemed important in incipient divergence (Coyne and Orr 2004) and apparently maintain genetic divergence in willow warblers (*Phylloscopus trochilus*) and yellow-rumped warblers (*Setophaga coronata*) complex in the absence of strong assortative mating (Bensch et al. 2009; Brelsford and Irwin 2009). Therefore, future research on the incipient divergence blackcaps should also consider postzygotic isolation as a potential mechanism driving divergence.

### Genetic diversity along the migratory divide

Our study on blackcaps differing in wintering origins found significant differences in genetic diversity along the migratory polymorphism in southern Germany. Migrants following the recently established northwestern route (Berthold et al. 1992) showed higher levels of inbreeding (*F*_IS_) and significantly lower individual genetic diversity, denoted as overall heterozygosity and mean d², compared with the two clusters of SW migrants that follow the traditional south-western route. These findings are consistent with the new route having evolved only recently, involving far fewer individuals than the SW route, and document thus for the first time that the genetic structure of the NW-migrating population deviates from that of SW-migrating populations.

At present, it remains unknown whether the lower heterozygosity we found among the NW-migrating blackcaps might affect their future adaptive potential. In general, low levels of heterozygosity are linked to lower population fitness (Reed and Frankham 2003) and also to the ability of populations to respond to selection (i.e., evolvability, Houle 1992). On the one hand, moderate inbreeding might constrain the evolvability of traits with strong additive genetic backgrounds, whereas on the other hand, it might assist the evolvability of traits with a nonadditive genetic background (Cheverud et al. 1999; Zhang et al. 2004; Van Buskirk and Willi 2006). Interestingly, life-history traits (e.g., timing of breeding) are known to have a strong nonadditive genetic background (Merilä and Sheldon 1999, Teplitsky et al. 2009) and are also important targets of selection in migratory birds (Both et al. 2006; Hedenström et al. 2007). Moreover, both the expression of the underlying genetic variance in life-history traits and the selection acting on them often depend on environmental conditions (Husby et al. 2011). However, our analyses are based on the variation in microsatellites and therefore on (neutral) genetic markers that generally (1) reflect only a very small portion of the genome and (2) are known to be poor indicators of adaptive genetic differences (Reed and Frankham 2001). Hence, further investigation of the evolutionary potential in our study population initially requires that quantitative genetic information of respective traits is measured directly.

### Multiple founder effects following the migratory divide?

The new migratory behavior in southern German blackcaps probably evolved within only a few decades from preexisting variation for migratory directions in central European blackcaps (Berthold et al. 1992; Helbig 1996). The newly evolved migratory route entails restricted gene flow between sympatrically breeding populations using different wintering quarters (Rolshausen et al. 2009). Assuming that the population genetic differences along the migratory divide can be explained by a recent colonization event (i.e., “founder effect,” Nei et al. 1975), two nonmutually exclusive hypotheses can be applied: The founding event(s) could have arisen (1) from within the local breeding population but also (2) from multiple contributions of more distant populations of blackcaps that also adopted a northwestern orientated migration (Helbig 1996). In favor of the first hypothesis, we found elevated inbreeding (*F*_IS_) and significantly lower genetic diversity (heterozygosity) for the NW migrants. However, NW migrants were on average genetically more distinct from each other than SW migrants (i.e., higher genetic distances within the third *δ*^2^H cluster), which did not differ genetically between isotope clusters (Fig. 3). Moreover, the bottleneck analysis of heterozygosity excess corrected for allelic diversity (Cornuet and Luikart 1996; Piry et al. 1999) did not yield evidence for a recent severe population bottleneck in the NW migrants. As such, we hypothesize that multiple founder events occurred as this hypothesis is consistent with a more diverse genetic background of individual NW migrants.

Both scenarios, introgression from more distant populations of northwest migrating blackcaps, as well as a founder event from within the local breeding population require incipient reproductive isolation and therefore asymmetric admixture in sympatry along the migratory divide to maintain the genetically based differences in migratory behavior (Berthold et al. 1992; Helbig 1996). Our admixture model in STRUCTURE that assumed three underlying populations to match the number of *δ*^2^H clusters (Fig. 2) found significant asymmetric admixture on neutral genetic markers related to the wintering areas of blackcaps (Fig. 4). Migrants from *δ*^2^H cluster 3 (i.e., NW migrants) were on average assigned with higher confidence to one of the three simulated genetic populations, that is, they had a higher probability of pertaining to a specific genetic cluster, as denoted in higher residual assignment coefficients (Δpr). Moreover, the within-cluster fractions of the third genetic population decreased along the N-S gradient from 36% in *δ*^2^H cluster 3 to 14% in *δ*^2^H cluster 1, a result which is strongly confirmed by examining the extremes of the *δ*^2^H spectrum and the correlation between assignment scores and individual *δ*^2^H values (Fig. 4). These patterns suggest that the migratory divide in southern German blackcaps has a significant impact on population genetic dynamics in sympatry. Yet, given the low overall genetic differentiation in the studied population (*F*_ST, N vs. S_ ≤0.008, for details see Rolshausen et al. 2009) along with nonequilibrium population dynamics due to the newly established NW route, the STRUCTURE simulation might not be a powerful tool to resolve the actual population structure at this early stage (see Pritchard et al. 2000). Still, our analyses motivate the hypothesis that NW migrants from foreign populations are more successful in introgressing the *δ*^2^H cluster 3, already containing NW migrants, and that this introgression could therefore be facilitated via assortative mating based on wintering origins and migratory strategies.

## Conclusions

Our analyses show a positive relationship between individual wintering origins and population genetic structure in sympatrically breeding blackcaps from southern Germany. The migratory divide of SW- and NW-migrating birds strongly contributes to this genetic structure, but it is also present within SW migrants. Moreover, individual differences in migratory behavior relate to differences in admixture among groups of blackcaps with distinct migratory behavior. We also found support for the hypothesis that the establishment of the migratory divide in southern German blackcaps may have involved multiple founder events that may have arisen not only from within the local breeding population but also from more distant blackcap populations also migrating in a northwestern direction. Taken together, our study indicates that individual migratory behavior influences genetic structuring in sympatric populations of migratory birds that show weak migratory connectivity. In general, this mechanism might be an important link between the genetic basis of migratory behavior and (1) the translation of environmental change into adaptive population genetic change (e.g., Pulido and Berthold 2010) as well as (2) the establishment and maintenance of migratory divides (e.g., Bensch et al. 2009; Brelsford and Irwin 2009). A potential proximate mechanism facilitating the genetic structuring in our study population is spatial divergence caused by differential microhabitat selection. We did not find evidence that individual arrival times varied consistently according to migration distances. Although differential arrival times of populations with distinct migratory routes have been discussed in the incipient population divergence of blackcaps in southern Germany (Bearhop et al. 2005; Rolshausen et al. 2010), the null model by Rolshausen et al. (2010) suggests that temporal isolation can maximally explain part of the incipient isolation and that other mechanisms are likely to also contribute to it. Hence, the results from our territory analyses are encouraging that future research on microhabitat selection in relation to migratory behavior in birds will provide more detailed insights into micro-evolutionary dynamics within populations responding to contemporary changes in selection.
